# A Reasonable Alternative System for Searching UAVs in the Local Area

**DOI:** 10.3390/s22093122

**Published:** 2022-04-19

**Authors:** Marek Češkovič, Pavol Kurdel, Natália Gecejová, Ján Labun, Mária Gamcová, Matúš Lehocký

**Affiliations:** 1 Faculty of Aeronautics, Technical University of Košice, Rampová 7, 041 21 Košice, Slovakia; marek.ceskovic@tuke.sk (M.Č.); pavol.kurdel@tuke.sk (P.K.); 2 Faculty of Electrical Engineering and Informatics, Technical University of Košice, Letná 9, 042 00 Košice, Slovakia; jan.labun@tuke.sk (J.L.); maria.gamcova@tuke.sk (M.G.); 3 Slovak Air Force 46th Transport Wing, 901 01 Malacky, Slovakia; matus.lehocky@mil.sk

**Keywords:** drone, inverse doppler VHF omnidirectional radio range, position indicator, UAV, UAV tracking

## Abstract

UAVs, used for professional purposes, often intervene in unfamiliar terrain and challenging conditions. Unlike recreational UAVs, such professional and specialised UAVs are very expensive to develop and operate, and their value is not negligible. Due to the nature of operations in an unknown or dangerous environment, there are also situations with forced interruption and termination of the flight mission or a collision with the environment. Locating a lost vehicle presents a new challenge for UAV operators. The possibilities of today’s localised commercial aircraft in distress (COSPASS/SARSAT systems) are undesirable for selective special-purpose drones. The optimisation of the location in the event of an emergency or catastrophic landing may be justified by a social or other condition, where the user wants to search for the device by a system other than the one experienced for rescuing people, ideally on their reserved frequencies. The article proposes a new approach to solving the problem based on the design of a terrestrial localisation system based on the methods of processing and correlation of the obtained data by the physical principle of the Doppler effect and its own system adaptation. This creates an innovative concept of a targeting system based on the broadcasting of distress (VHF) signal by crashed UAV. This signal is captured and evaluated by the IDVOR system, making it possible to determine the direction in which the searched UAV is placed. In order to determine the difference between standard targeting systems of the UAV, which use information about position (exact coordinates (x,y,z)), the IDVOR system is able to determine direction, independent of other systems in every “enemy” or “inhospitable” territory.

## 1. Introduction

The use of unmanned aerial vehicles (UAVs) is currently widespread. We do not mean only their primary use for commercial or leisure activities; instead, we increasingly encounter unmanned technology in strategic applications, such as search and rescue humanitarian or military use [1,2,3,4]. Regardless of the current use of the UAV, the loss of such equipment would represent significant financial damage for the owner (operator). In addition, in the case of military or other strategic use, damage/loss to the UAV could result in much more significant loss, such as loss of collected data, damage or seizure of useful equipment carried onboard the UAV by an unauthorised person, etc. This could lead to a fatal failure of the ongoing mission, which could jeopardise the safety of individuals. Therefore, it is necessary, especially today, to pay attention to the correct and efficient tracking of a moving unmanned target not only during its movement but also in the event of its accident/failure/loss in the field. Although today’s UAVs are equipped with a number of protection features, including the autonomous return, there are still situations where the device needs to be traced in person [5,6,7].

Operations that require the deployment of unmanned equipment are most often missions that take place in unfamiliar or difficult terrain. This is often in an area that is not accessible by standard means, is not mapped (intentionally or unintentionally), or is an area with GNSS signal outages/interruptions. For this reason, the exact location cannot always be determined. In the event of a UAV failure or inability of the UAV to return to its “home” (initial) position, in the event of an accident or other loss (shooting down) of the UAV in such an area, it is essential to obtain to the location as soon as possible. Otherwise, the collected data that carry useful equipment, or even parts of the UAV, could be misused by the other party [8,9]. Since this is a specific, local area in which the UAV operates, it is possible to create alternative ways of tracing and detecting a particular target [10].

Currently, a variety of options for tracking a remote air target are offered. From publicly available small compact devices designed to track owned devices (e.g., Apple AirTag, SmartTag, etc.), which operate on the principle of using GPS signal and location with the help of other devices compatible with this tracker [11,12], to EPIRB (Emergency Position Indicating Radio Beacon) emergency transmitters in maritime transport or ELT (Emergency Locator Transmitter) used in aviation [13,14,15]. Searching for drones in the spectrum’s optical, thermal, infrared and electromagnetic sides has also been a long-studied problem. The mentioned methods of emergency/rescue location are connected by the fact that it is a broadcast on a publicly available and known frequency, or a search for a target or a missing person, using GPS signal and information obtained from compatible mobile devices located near the object [16,17]. All of these methods can be exploited not only by the party looking for the UAV but can be abused by an unwanted counterparty. Therefore, creating an alternative search method is crucial, the so-called “reasonable alternative system”. Its use would be primarily targeted at tracking the chosen target, in our case, the UAV, after the incident or accident on its own, predetermined frequency, which would be known only to verified persons authorised to search. The effectiveness of the tracking system would mean a significant increase in the efficacy of any intervention that would take place in an inaccessible area or in an area where there is a tactical justification for the use of UAVs.

The currently used methods of UAV detection are most often used in terms of counter-drone technology. Their purpose is to detect the activity of the UAV, alert the authorised persons to the breach of space and, if necessary, take action against such a device, from intentional disruption of communication, forced landing, taking control to the malfunction/destruction of the UAV. These technologies work in four steps—detection, recognition, monitoring and alert/intervention. They use the methods listed in Table 1 to detect UAVs.

The growing demand from manufacturers and operators of UAVs for SAR (Search and Rescue) or military applications has led the authors to consider an alternative way to locate UAVs, not just in-flight tracking [18]. For this reason, from Table 1 above, we can categorise our IDVOR targeting system into the group of RF analysers and RDF (Radio Direction Finders). For the system’s greatest possible mobility and simplicity, we did not use triangulation (i.e., at least three receivers) but one mobile system using the mentioned principle of the inverse DVOR function. Thanks to that, we can target the direction from which the transmitted “emergency” high-frequency signal from the fallen UAV propagates. We did not obtain a specific position (exact coordinates (x, y, z)) as that would require three IDVOR receivers. Instead, in our case, we determined the direction from which the signal is carrying/transmitting.

**Figure 1:**
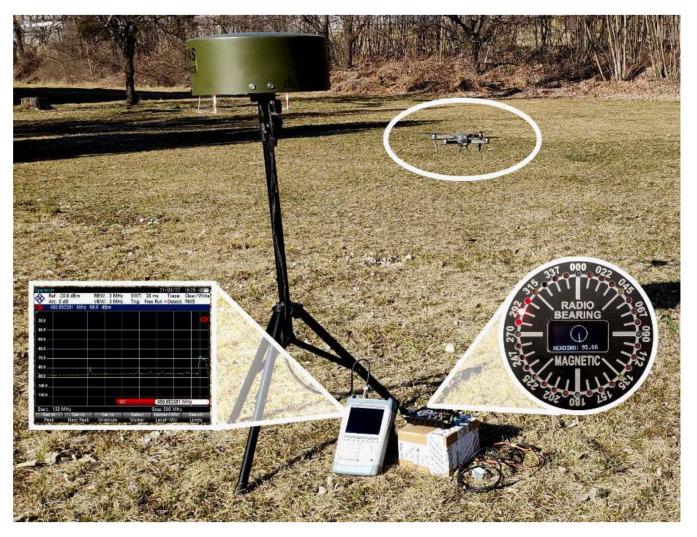
The model situation of UAV localisation with IDVOR system in the local area. Spectrum analyser is used for signal detection, and the LED signalisation on the control panel presents the direction of the tuned signal (UAV).

The presented article offers an alternative way of searching/finding the lost UAV device in a way that would be available only to an authorised user. The ground targeting system considered in the article was not only designed but also experimentally tested. The principle of operation of the targeting/tracking device is ideologically derived from the approach navigation system DVOR (Doppler VHF Omnidirectional Radio Range), which has long been used in aviation [19,20,21,22]. The undeniable advantage of this newly built system for the method of localisation is, above all, the speed of UAV search and precise directional navigation to this point in unknown terrain without the need to use a map or GPS device (Figure 1). 

The idea of using the DVOR system as a template for a new tracking system was based mainly on the fact that it is a system with low energy requirements in detecting and locating the position of the radio signal source. The proposed system itself does not carry any geographical location information, and it does not even use any location detection subsystems. It transmits any (user-defined) high-frequency signal to the space [23,24]. Such a high-frequency signal, with a frequency known to the user, can be radio-focused with different accuracy. The accuracy of the focus depends on the design of the system and the subsequent interpretation of the result from the radio signal source (in the case of the DVOR aeronautical system, it would be the heading angle to the radio station) and its direction. In the case of focusing the transmitted high-frequency signal by two or more devices (terrestrial receiving stations) located at more distant places, it is possible to more accurately determine the 2D position of the crashed UAV device. However, this method requires additional equipment and additional personnel.

**Figure 2:**
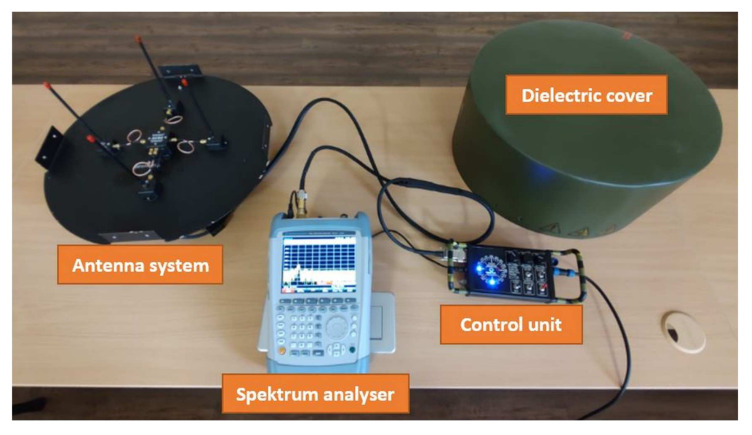
IDVOR radio signal targeting system designed for portable operation.

The designed ground targeting system (Figure 2) works on the inverse principle of the DVOR radio navigation device with a more precise determination of the heading angle and the guideline. The device working on the principle of IDVOR (Inverse Doppler VHF Omnidirectional Radio Range) provides localisation of the lost device with an accuracy of 5°. The advantage is the possibility of creating a compact version of the tracking device, which allows installation on a land, mobile or marine platform. This makes the tracking system easily portable, allowing easy transport and commissioning by one person within 5 min.

## 2. Materials and Methods—Empirical Design of the IDVOR Targeting System

The proposed IDVOR system, which is intended as a justified alternative sight of the emergency signal, is, in the present case, situated in radio sighting in the VHF and UHF bands [25,26]. In this band, the operator can choose his own frequency range with an alternative that increases the signal’s immunity to interference. If the possibilities of the terrestrial system for searching UAVs at other frequencies are taken into account, i.e., the broadband of the antenna system, in that case, it is necessary to consider protection against the influence of radio targeting disturbances.

The influence of the environment, which cannot be neglected at these wavelengths, is the propagation of the ground wave, where the attenuation mechanism is applied, in the case of obstacles to reflection and subsequent dispersion (scattering after reflection). It should also be noted that with increasing signal frequency, diffraction (signal bending) does not occur. With regard to these facts, it can be stated that at the point of reception (using the pair Receiver IDVOR—HF emergency transmitter on the UAV), there is the highest quality reception at the point of maximum intensity of the electromagnetic field. That is, in the interference caused by the direct propagation of the surface wave and its reflections. Based on this knowledge of radio wave propagation, the range and possible targeting of lost/crashed UAVs are best with using the antenna elevation option. This is performed by elevating the transmitting or receiving antenna.

During the creation and implementation of the prototype device itself, completion of individual sub-parts of the IDVOR system, elimination of deficiencies and subsequent experimental testing of product prototypes, care was taken to ensure that TSO and ISO standards were complied with [27,28]. 

The first step to the successful practical implementation of the system is to define and clarify the issues related to the propagation of radio waves, from which the sequence of steps is derived.

### 2.1. The Influence of the Environment on the Level of the VHF Signal at the Point of Reception and the Problem of VHF Signal Propagation for the Purpose of UAV Targeting

In the theoretical analysis of the proposed IDVOR system, the VHF radio wave signal propagation mechanism was also considered [29,30]. The propagation of a radio wave in the lower layer of the troposphere above the flat earth’s surface is considered when the position of the UAV transmitter is at a higher altitude and the IDVOR receiver is located just above the earth’s surface. In this way, both a direct wave and a wave reflected from the earth’s surface enter the receiver from the transmitter. With this wave propagation mechanism, the effective value of the field strength *E_ef_* can be expressed, which is based on Kirchhoff’s solution of Maxwell’s equations [31], with a proper interpretation of the cosine theorem, defined by Equation (1).

(1) $$E_{ef}=\frac{173\sqrt{1+{\left|R\right|}^{2}+2\left|R\right|cos(𝜃+\frac{2𝜋}{𝜆}\Delta r})}{r}\sqrt{PD}$$

where *R* is the Fresnel wave reflection factor from the ground, *θ* is the phase shift of the wave reflected from the ground, *r* is the distance between the transmitter on the UAV receiver IDVOR, Δ*r* is the path difference between the direct wave and the wave reflected from the ground, *λ* is the wavelength, *P* is the radiated power of the transmitter and *D* is a factor of directionality of the transmitting antenna.

In the case of targeting a moving object (UAV) whose flight altitude is much less than the distance between its transmitter and receiver, the original relationship can be adjusted. Under this condition, the reflected wave from the ground has a tangent character, and its value is proportional to −1. This means that the amplitude of the reflected wave has a maximum value, and its phase shift *θ* is 180°. This fact allows us to simplify the previous Equation (2).

(2) $$E_{ef}=\frac{2.8\sqrt{PDH_{t}H_{r}}}{r^{2}}$$

where *H_t_* is the height of the transmitting antenna, and *H_r_* is the height of the receiving antenna.

Based on practical experience, it can be stated that in addition to the height of the transmitting *H_t_* and receiving *H_r_* antenna and the radiated power *P*, the electrical properties of the earth’s surface and the shape of its relief also have a significant effect on radio wave propagation in the case of VHF. The earth’s surface negatively affects the reflected radio wave due to diffraction, reflection and scattering.

This means that there is a maximum attenuation of the direct surface wave by the reflected wave, and the phase shift can be calculated according to Equation (3).

(3) $$\theta =\pi +\frac{2\pi }{\lambda }\Delta r$$

where Δ*r* is the path difference; the smaller the resulting value of$\;\frac{2\pi }{\lambda }\Delta r$, the more the different propagation modes (signal reflected from the ground and rectilinear propagating signal) are compensated for each other. For this reason, income is declining, mainly due to other external factors. The field strength *E*_0_ at the receiving point, which is triggered by the transmitting antenna, can be expressed by the following Equation (4).

(4) $$E_{0}=\frac{\sqrt{30P_{out}}}{D}$$

where *P_out_* represents the radiated power of the targeted transmitter, it is deducted by the attenuation of the high-frequency line, to which the gain of the transmitting antenna is added. In a given relationship (4), the variable *D* represents the distance between the transmitter and the receiver.

When searching for a target, it is possible to determine only the theoretical distance *d*, which can be focused on; this is expressed by Equation (5).

(5) $$d=\frac{10\left(route\_attenuation-20\log\left(f\right)-32.5+2G_{Tx}\right)}{20}$$

This calculation (5) is also burdened by various radio wave propagation conditions up to the final signal processing. In such a case, the route attenuation in one-way propagation can be expressed according to Equation (6).

(6) $$route\_attenuation=32.5+20\log\left(d\right)+20\log\left(f-2G_{Tx}\right)$$

where *G_TX_* is the gain of the transmitting antenna and *d* is the theoretical distance over which the target can be targeted (the crashed UAV, which in our case, we are trying to find), and *f* is the frequency.

Based on practical experience, it can be stated that in the case of VHF, in addition to the height of the transmitting and receiving antenna and the radiated power, the surface of the object itself (its electrical parameters) also has a significant effect on the propagation of the wave behind the obstacle. This is because it is on the surface of the object that is the obstacle that the diffraction, reflection and scattering of the wave occur. This allows partial propagation beyond the terrain barrier, where the field strength that arises at the reception point is then taken into account. This value is presented by the reception intensity diffraction equation, the simplified form of which is described in the following Equation (7).

(7) $$E_{ef}=\frac{173\sqrt{PD}}{r}F$$

where *F* is the attenuation factor of the earth’s surface. However, a closer mathematical expression of this attenuation is very challenging. Therefore, from a practical point of view, this value is often defined graphically, based on knowledge of the electrical properties of the earth’s surface and the shape of its relief. These interactions of the radio wave with the material environment can also positively affect the propagation of the waves. It allows it to propagate, even for a low terrain obstacle.

Other factors that affect the spread are the temperature and humidity of the environment. These factors are also complicated to quantify, but their impact is not negligible in long-term observation. The conclusion of the studies [32], whose task was to investigate these effects, is that the signal strength is inversely proportional to atmospheric temperature and directly proportional to relative humidity. The effect of relative humidity and temperature is higher on cloudy days than on sunny days. This applies to the VHF band. These changes were observed in long-term (several days to several months) measurements. In our case, targeting the UAV is a short-term process, so we can consider these influences constant, we neglect them.

In the case of a mountainous area in which the searched UAV may be located, determining the field strength at the receiving site is very complicated. The hills form different angles with the ideal plane, and without specific and geographically accurate knowledge of the terrestrial surface at a given location, the calculation or prediction of intensity is highly inaccurate. In practice, we can mention cases where there was a random superposition of reflected direct waves, which caused interference between all propagation modes. In this case, two scenarios can occur, an increase in the field strength at the receiving point or a decrease. For this reason, it is not possible to clearly determine whether or not direct focus will be ensured even in very mountainous terrain.

However, it can be stated that the propagation behind the terrain obstacle is sporadic in the VHF band. The field strength behind such an obstacle is often so low that commonly available receivers cannot process such a signal. Respectively, in high-frequency circuits (antenna, antenna selector, HF line), there is attenuation, and the intensity of the signal intended for processing is below the threshold level. For this reason, the receiving stations are located in an area with a high altitude above the surrounding terrain.

A deeper analysis of propagation from a source of high-frequency radiation (in our case, a transmitter onboard a UAV) to a receiver (in our case, an IDVOR receiver) is only possible for specific place cases and known quantities. In the case of a low level of signal processing in the receiver, this information appears insignificant compared to the noise. The value level coincides or oscillates with the noise level; this manifests as sporadic, intermittent reception. The effect of atmospheric disturbances can also manifest itself. These faults have the most significant effect on short waves; in the case of VHF/UHF, it only applies at close distances. In the case of atmospheric discharges, broad-spectrum disturbances in radio transmission occur. Their spectrum affects the VHF/UHF band at a lower level than HF and VLF. The centre of such a spectrum of radio disturbances, the impulse produced by atmospheric discharges, is around the level of 800 kHz.

In the VHF/UHF band, interference disturbances occurring at a possible targeting source are more frequent, for example, static discharges arising at the surface of the targeting device/UAV. The influence of intentional or unintentional interference (for example, industrial interference) can generally be assessed as interference from the point of view of the intensity of the electromagnetic field. At the reception point, it must be ensured that the parasitic high-frequency signal does not enter the receiver and is not processed incorrectly. This means that it is necessary to prevent its processing instead of the desired signal or avoid mixing with the desired signal, the emergence of intermodulation. The most common unintentional interference is caused by a signal from an adjacent channel, i.e., channel spacing. This decreased in VHF aeronautical communications thanks to the ability to design a receiver with sufficient selectivity in the intermediate frequency part of the receiver and the need to obtain a more significant number of communication channels. The receiver’s selectivity is given by the ability of the used filter to filter out unwanted components and pass only the required spectrum width. The problems that would occur when changing the attenuation characteristic of such a filter are manifested by intermodulation and distortion of the low-frequency part.

Another type of interference that occurs is interference at the receiver’s IF frequency. This interference may be used intentionally for tactical reasons, or it may occur unintentionally. This condition occurs when a strong transmitter operates on the IF frequency of the receiver used; its signal can penetrate through parasitic paths into the IF frequency of the receiver, where it is processed together with the required useful signal. This can cause interference, which ultimately affects the low-frequency component. For this reason, it is not possible to target the required high-frequency signal generated by the crashed/traced UAV. Such unwanted penetration is caused by insufficient shielding, the resulting skin effect on shielding or insufficient selectivity of tuned circuits. The prevention of such interference lies in the use of debuggers. 

Some of the above-described problems with possible interference could be avoided by settings on the IDVOR prototype control panel and antenna system positioning.

### 2.2. Design of the Targeting System IDVOR

As mentioned in the introduction, the idea of the new search system is based on the concept of the DVOR system (respectively, VOR), which has been used in aviation since the 1950s, thanks to which its reliability and robustness were verified.

**Figure 3:**
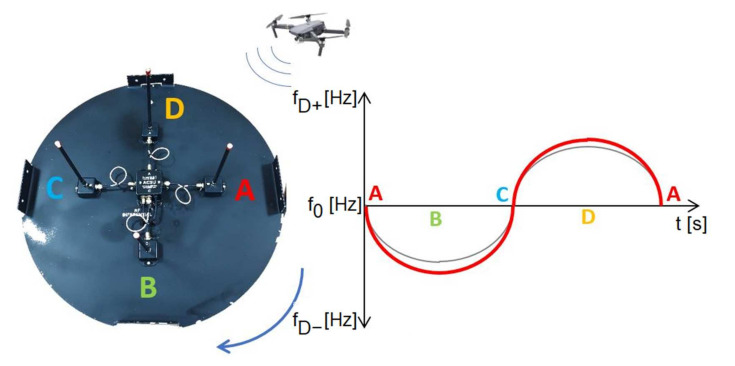
Graphic representation of the focus principle using the IDVOR system. On the graph, the grey line represents switching signal, and red line represents received signal.

Similar to the DVOR system, our proposed IDVOR (Inverse Doppler VHF Omnidirectional Radio Range) system uses the Doppler principle of direction-dependent signal generation. In our proposed system, the antenna system is switched at high speed by a multiplexer. With four antenna switches, the system appears to the observer as a single antenna rotating in a circle with a switching frequency of approximately 500 Hz, i.e., 500 rpm. The received frequency has an increasing character when approaching the point on the circle that is closest to the transmission source. This means that the frequency of the received signal increases. The received frequency decrease if the antenna moves away from the source (Figure 3).

**Figure 4:**
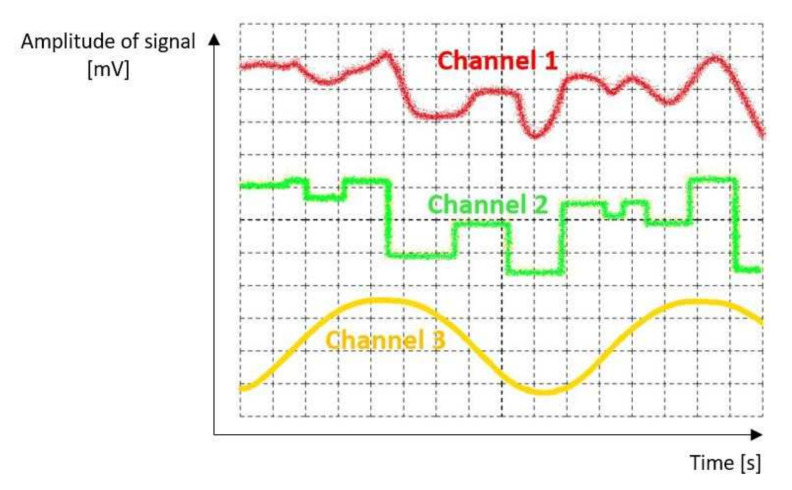
Sample of graphic courses of Doppler signal (after “digitization”), clock and switching signal. Channel 1 (noise + useful audio signal + Doppler shift) represents an audio signal processed from the receiver, which is affected by Doppler shift. Channel 2 (useful audio signal + Doppler shift after modulation and noise filtration). Channel 3 (clear Doppler shift signal)—signal after low-pass filtration.

The principle of operation of the IDVOR system is that after capturing and receiving the high-frequency signal and its demodulation, the demodulated signal is adjusted to sinusoidal by means of filters. The phase ratios in this signal compared to the support signal from the multiplexer are decisive. At point A (Figure 3), the received frequency is identical to the support (reference) signal; the relative movement of the antenna is not recorded, so the received signal is in phase with the support (reference) signal. By switching the antennas with a multiplexer clockwise, the antenna makes a relative movement to the signal source; it moves away from the source. For this reason, the received frequency is lower, and a shift is noticeable. The Doppler shift reaches the highest value at point B, so the frequency is the lowest. As the antenna begins to make a relative approach to the signal source, the frequency increases. At point C, the frequency is again equal to the frequency of the targeted transmitter, as it makes no movement relative to it. Analogously, positive frequency values are also acquired. From point C to point D, the antennas perform an apparent movement again, but back towards the source of the targeted signal, the frequency increases again. The frequency decreases again to the source (reference) frequency from point D to point A. After connecting the radio receiver to such an antenna system and tuning it to the desired frequency, the switching frequency is audible as a sound with a frequency of 500 Hz after demodulation. This rotational motion can be plotted as a sine function (Figure 4). The timing must be accurate and, at the same time, the same for the antenna multiplexer and the rest of the targeting system [19,20,21,22,23,24,25].

The measurement consists of adjusting the received signal by filters to the form of a sine wave and detecting the zero state of the phase in the clock rhythm of the multiplexer. The process continues by comparing the detected signal with the course of the switching signal, which switches the antennas and, at the same time, generates a support (reference) signal. It can be used to analyse the received signal to measure the Doppler shift. Thanks to this step, we obtained the relative direction between the source and the centre of the antenna system. 

The start of the measurement is shown in Figure 3: the start of the multiplexer; antenna connection A; switching antennas A, B, C, D by an antenna switch with a specified switching frequency. In the example shown in said figure (Figure 3), the switching signal (indicated by the grey line) is in phase with the received signal (indicated by the red line). By crossing the *x*-axis at point A, the measurement is terminated, and a new one is started simultaneously; the result is equal to zero. Similarly, the address counter is started, in which the corresponding address is set in the elapsed time—LED, i.e., 0. This means that the direction to the source is in the direction of 0° (antenna A).

**Figure 5:**
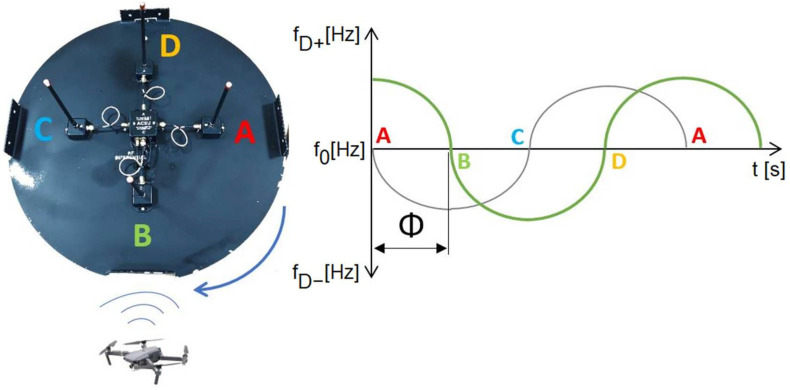
Graphic representation of the targeting principle using the IDVOR system for the case of targeting the next position. The *θ* represents phase shift.

The following figure (Figure 5) shows the movement in the direction of the source closest to antenna B.

The address counter is started, as mentioned above, always at the point of antenna A. By switching clockwise, antenna B is connected, which in this hypothetical case is closer to the source of the targeted signal. The signal is close to the nominal value of the frequency of the targeted source, while an address counter is built in at the point where the nominal frequency is reached (*x*-axis intersection). The address of the LED display segment is also defined at this point; in this case, it is 90°, which is the direction of the signal source.

The designed IDVOR device consists of three interconnected units. IDVOR antenna system, any radio station as a receiver and demodulator (YESU FT-817ND station and Rhode&Schwarz FSH 8 spectrum analyser were used for field testing) and IDVOR’s own evaluation device. The antenna system consists of four antennas, which are switched with a frequency of 500 Hz in a clockwise direction. The antenna system receives the high-frequency signal we need to target. At the same time, the antenna system contains an ACSU unit, which is an antenna switch controlled by a multiplexer of the evaluation device. By switching antennas with a frequency of 500 Hz at a specified radius and with an unknown direction of reception, we achieve at the output of the antenna system a received signal that contains a focused high-frequency signal with a frequency rise or fall. This change, i.e., the frequency increase/decrease, is the result of the Doppler effect.

**Figure 6:**
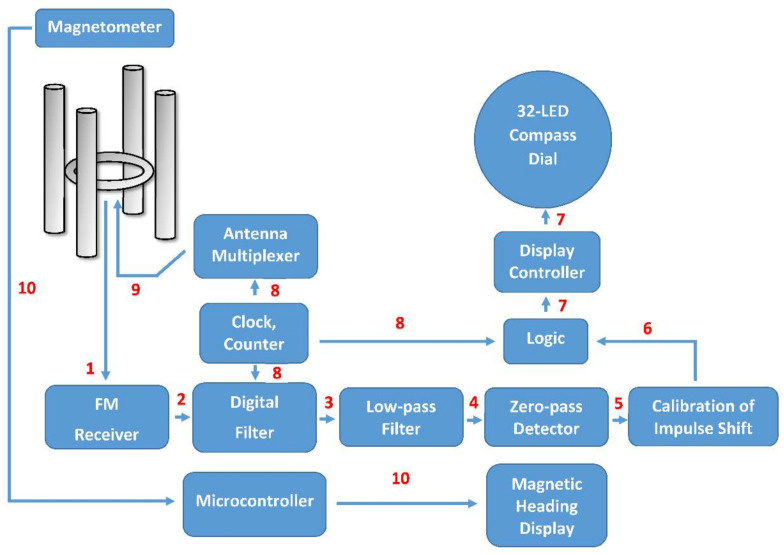
Block diagram of the IDVOR system.

The signal processing flow chart of the proposed system (Figure 6) can be described as follows:

1. The targeted high frequency modulated signal is received by the first antenna (from four antennas), and then it is processed to the receiver for demodulation. After that, the second, third, and fourth antenna receives this same signal and pass it to the receiver for demodulation;
2. After demodulation, the low-frequency audio signal containing Doppler shift part is passed to the digital filter;
3. The outcome of digital filtration is an integrated waveform with very low bandwidth;
4. The low-pass filter cuts out unwanted noises and peaks. The “clear Doppler sig-nal” is extracted from the previous waveform after a sinusoidal clock reference adjusts the centre frequency signal;
5. Zero-pass detector waits for the exact moment when there is no (zero) phase shift. After detection, the signal is passed to the presettable pulse shift delay function;
6. The pulse can be shifted, which allows calibration of the proper value of the detected radio signal bearing. Moreover, a logic that allows “freezing” of LED compass display is present;
7. The information about the heading is processed to the display controller and illuminates the appropriate LED;
8. Reference synchronisation signal (500 Hz) starts the counter and initialises the algorithms;
9. Clock signal drives multiplexer switches one from four antennas at a time and connects them to the receiver. The rotation is clockwise. Each cycle starts from antenna A, which heads towards the north for reference;
10. Communication between magnetometer and controller runs on an I2C data bus. The output is a magnetic heading.

### 2.3. Method of Calculating IDVOR System Parameters

The overall design of IDVOR was based on the required equipment parameters and technical capabilities of the manufacturer. The pursuit of the highest possible compactness and mobility of the IDVOR device was the reason why a four-element antenna system was chosen—an arrangement of four monopoles on artificial ground. In such an arrangement, the minimum distance of the individual elements from each other must be kept. This distance should not be higher than λ/4. The wavelength at the intended test frequency of 430 MHz for targeting the crashed (lost) UAV corresponds to *λ* = 0.69 m/4 = 172 mm. The Doppler frequency was set at 500 Hz, based on the available component base. From the possibilities of the used multiplexer, from which the switching frequency is derived (Equations (8)–(12)), it was set to 511 Hz [29,30]. 

The calculation of IDVOR system parameters is as follows:

(8) $$f_{r}=\frac{f_{D}\times 1879.8}{Rf_{c}}$$

where:

*f_r_*—rotation frequency;

*f_D_*—Doppler frequency (Hz);

*f_c_*—frequency of the targeted signal (MHz);

*R*—radius of antenna rotation (minimum *λ*/8 (inch)).

(9) $$f_{r}=\frac{f_{D}\times 1879.8}{Rf_{c}}=\frac{500\times 1879.8}{4.274\times 430}=511\;Hz$$

(10) $$f_{D}=\frac{rf_{c}}{c}$$

where:

*f_D_*—Doppler frequency;

*f_c_*—frequency of the targeted signal (Hz);

*ω*—angular speed of rotation of the antenna;

*c*—the speed of the light;

*r*—radius of antenna rotation (m).

(11) $$f_{D}=\frac{rf_{c}}{c}=\frac{2909\times 12\times 430\times 10^{6}}{299792458}=500.69\;Hz$$

so

(12) $$\varpi =2f_{r}=2463=2909\;rad.s^{-1}$$

### 2.4. Calculation and Design of the Antenna System of the IDVOR

**Figure 7:**
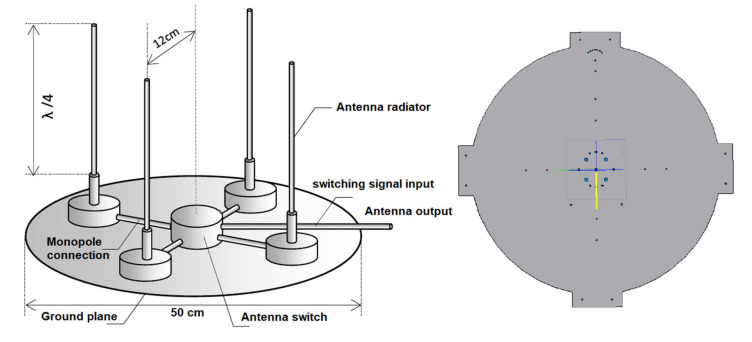
Model design of the antenna system and ground plane of the IDVOR targeting device.

The antenna system design for IDVOR (Figure 7) must consider all aspects of use as a mobile tracking/targeting system. As mentioned, the antenna system is structurally based on the principle of a quadruple monopole [33] with a wavelength λ/4, arranged in a cross on an artificial ground, formed by the aluminium sheet. The 50 cm diameter sheet metal itself was cut with a CNC milling machine. The size was deliberately chosen as it is necessary to provide a sufficient counterweight of 12 cm from the monopole and, at the same time, be 12 cm from the centre (Figure 7).

The monopole is based on a supporting frequency of 430 MHz and tuned to its resonance. Each of the four monopoles is adapted with a 1 kΩ resistor connected in parallel between the monopole and the artificial ground. This is due to adjusting the output impedance to 50 Ω and for the discharge of any static charge.

(13) $$\lambda_{M}=\frac{c}{f}$$

Calculation:

(14) $$\lambda_{4}=\frac{\frac{c}{f}}{4}\;k=\frac{\frac{299.792}{430}}{4}\;0.92=0.16\;m$$

The active length of the monopole is 16 cm (Calculation (14)), taking into account the element thickness of 8 mm. Structurally, each monopole is mechanically connected to artificial ground via an insulating pad, which is formed by the cover of the HAMMOND HM-1551 box. The box contains a double-sided printed circuit board on which the PIN diode D1 RN731VTE-17 in the SOD 323 housing is mounted. The SMA connector, which connects an RG316 coaxial cable with the same length of 15 cm, to the corresponding SMA connector ACSU (Antenna Central Switching Unit) was used for the connection. The box, which serves as a base, is bolted to the artificial ground. In the centre of the underside of the artificial ground is mounted a bracket for attachment to a tripod or mast with a diameter of 35 mm. The HAMMOND HM-1590AF aluminium distribution box is also mounted on the bottom, in which the connectors, the TNC female for RF signal output marked RF DIFFERENTIAL and the data connector, socket type M12 marked SWITCHING DATA, with four wires for switching antennas and for connection of the I2C interface of the HMC 5983 magnetic compass sensor. The HMC 5983 magnetic field sensor in the HAMMOND HM-1551 box with the possibility of five-stage direction rectification is mounted on the front reference side, which corresponds to the antenna A mounting. In the central part of the monopole page, the ACSU antenna-monopole switch is located in the HM-1590L aluminium box. The monopole part of the artificial earth is covered with a sandwich dielectric fibreglass cover. All screws used on the antenna system are made of non-magnetic stainless steel, which prevents the effect of parasitic magnetic fields on the HMC 5983 sensor [31,34].

In order to create a directionally dependent Doppler signal with a frequency of 500 Hz, it would be necessary to rotate the antenna at 500 rpm, which is technically demanding and almost impossible. The principle of fast electronic switching was chosen, similar to the case of the standard DVOR aviation system. For electronic switching of antennas, was designed an electronic switching circuit based on PIN diodes.

### 2.5. Reference Compass for Accurate Targeting Using the IDVOR System

For the correct functioning of the designed targeting system IDVOR, it is necessary not only to capture the high-frequency signal, correctly demodulate and evaluate it but also to compare the reference. For real routing of the targeting source of the radio signal, it is necessary to know the current direction or orientation of the antenna system to know the orientation of the first antenna (antenna A). For this reason, IDVOR is equipped with an electronic compass that helps determine the direction of the magnetic north. The Honeywell HMC5983 sensor we chose is a temperature-compensated three-axis integrated magnetometer. The multi-chip module is designed for magnetic sensing at low, even threshold levels of magnetic field strength. It allows detection and determination of vehicle/UAV direction to the Earth’s magnetic field. Alternatively, it can be used as a position sensing element based on a change in another magnetic field.

### 2.6. Evaluation Device of the IDVOR System

**Figure 8:**
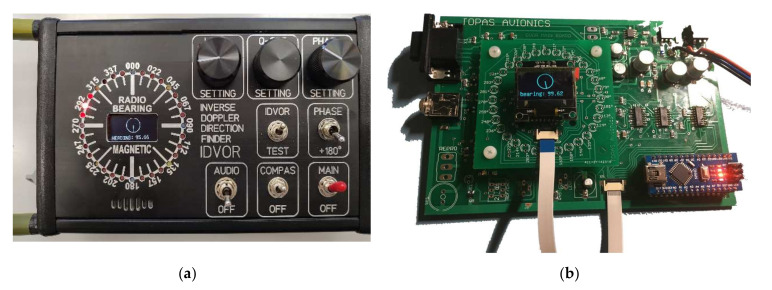
IDVOR control panel (**a**) and system board (**b**).

The device (Figure 8), designed to evaluate the received signal and direct the operating personnel to the high-frequency signal source (in our case, UAV localisation), has several functions. It switches antennas, shows the navigation compass, evaluates the received signal and displays the direction of target; tests the antenna system; and provides supply voltage for the circuits and components of the entire IDVOR targeting device. The evaluation device is structurally mounted in an aluminium box HAMMOND HM-1455-L. The components of the evaluation device are mounted on two double-sided printed circuit boards using SMD (Surface Mount Devices) technology (Figure 8).

The printed circuit boards were designed in the CAD/CAM EAGLE Layout Editor environment and made to order at the supplier. The larger printed circuit board, called MAINBOARD (dimensions 145 mm × 100 mm), contains all the circuitry needed for antenna switching and Doppler signal analysis. The smaller board (dimensions 60 mm × 60 mm) contains the IC7 4051 controller for the compass rose of the evaluation device, consisting of 32 LEDs. The compass rose allows the reading of directional information with an accuracy of 5.5° if two adjacent LEDs light up simultaneously. A separate reading provides a resolution of 11°. Course (directions) 000°; 090°; 180° and 270°are indicated by blue LEDs, course (directions) 045°; 135°; 225° and 315° are indicated by yellow LEDs. Red LEDs indicate other courses (directions).

The “LEVEL SETTING” controls are located on the front panel (Figure 8), and these are used to adjust the level of the input signal and signal’s peaks and stop scanning the direction. Another control element is “Q-SET SETTING”, which sets the bandwidth and thus the sensitivity of the Doppler signal input filter. “PHASE SETTING” is used to shift the reference signal phase, i.e., the directional setting of the rose calibration. The “PHASE 180°” switch shifts the phase of the reference signal by 180° to change and calibrate the direction of the antenna system.

The IDVOR device is activated by a switch (KNX-3) called MAIN. This is performed by switching from the OFF position to the MAIN position. Subsequently, the comparative digital magnetic compass is activated by the COMPAS switch. The AUDIO signal switch connects the audio signal input from the receiver to the miniature speaker, which is glued to the front panel below the compass rose. The IDVOR/TEST switch switches the function of testing the antenna system, and in the IDVOR mode, it performs the function of targeting radio signals.

The antenna system is connected to the IDVOR evaluation device via a D-SUB 15 PIN connector, which contains control outputs for switching antennas, an I2C interface for compass, supply voltage and outputs for control without the need to disassemble the device. To the right of the D-SUB connector is a female connector (3.5 mm stereo audio jack) for connecting the input of the demodulated signal from the receiver. There is a USB-B mini connector with a cover on the left side panel, which leads to the programming interface of the Arduino NANO development board microcontroller. This interface was retained for calibration or a possible magnetic compass firmware update. There is an AMPHENOL SP1310 connector on the right-side panel, through the pins of which a supply voltage in the range of +14 V to +28 V is supplied. The consumption of the entire IDVOR device is approximate 300 mA at +14 V.

For ergonomic handling and protection against damage to the device, the evaluation device is equipped with steel handles on the sides. In the rear part, the device is underlaid with rubber feet, and in the central region, a magnetic mounting mechanism with a ball pin from the 3M company is attached.

## 3. Results—Testing of IDVOR Equipment in Real Conditions

**Figure 9:**
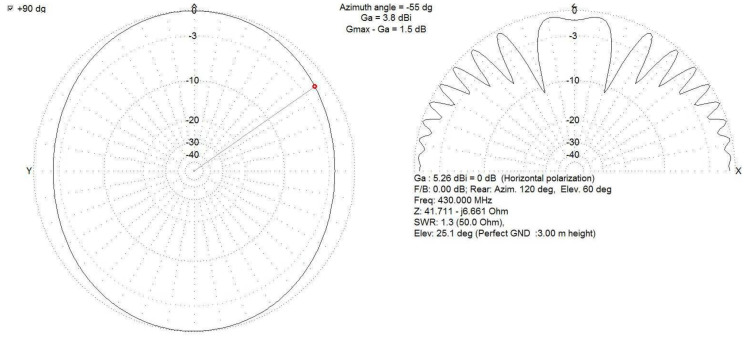
Simulation of the radiation characteristics of the antenna monopole of the IDVOR system both in horizontal and vertical planes.

Testing the IDVOR device in real conditions was preceded by a simulation verification of the radiation characteristics of the used antenna monopole (Figure 9). The simulation has proven that such a design will suit our application.

The initial testing of the IDVOR system took place in a closed room. The influence of the room was manifested by a problem with reflections and distortion of the direction of reception caused by obstacles; this condition was expected.

**Figure 10:**
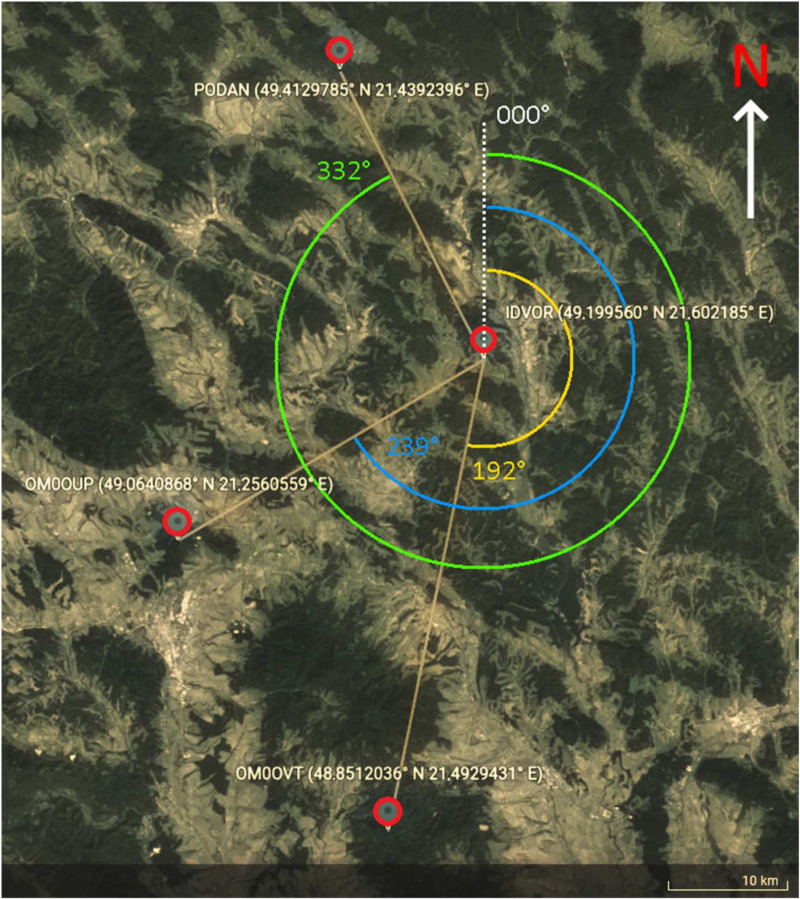
Location of testing points for evaluations of IDVOR.

Testing in real conditions (summary of the parameters is in Table 2, and a visualisation of the situation is in Figure 10) took place in the field with an elevation of 49.199560° N 21.602185° E at an altitude of 415 m a.s.l. After assembling the IDVOR set and placing the antenna at the height of 2 m above the ground, the IDVOR antenna system was oriented with a reference point to magnetic north according to the integrated comparison compass. Subsequently, the receiver was tuned to the frequency of the amateur radio converter OM0OUP, located at the elevation of Furnická Stráž near Prešov, transmitting on the frequency 439.1 MHz; this converter was chosen as the initial test point simulating a crashed UAV device. Another radio station remotely activated this test transmitter to the broadcast state; thus, the targeting testing began. The test transmitter was immediately targeted, and the direction displayed on the compass rose at 236°. The position of the test transmitter was verified using a mobile targeting device, which determined the course (direction) with a value of 239°.

**Figure 11:**
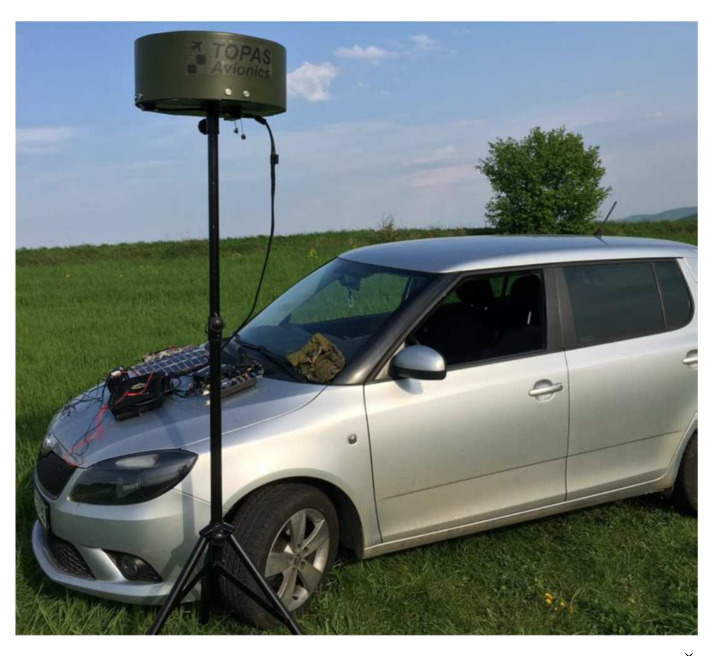
Exterior targeting station of the IDVOR system in the Šandal area.

The position of another tested OM0OVT transmitter, located in the Makovica area in Slanské Hills, was similarly targeted (Figure 11). The LED on the compass rose was lit on course 191°. The mobile targeting device determined the reference course at 192°.

Further testing took place on a flat meadow, in the centre of which the IDVOR was placed, and the position of the signal source was modelled by a moving YAESU VX-6R radio station (with a set transmission power of 250 mW) in a circle around the IDVOR antenna system. This testing was also successful.

Another attempt was targeting a transport aircraft flying at flight level 300. Its position was determined using an ADS-B receiver and software. Thanks to the aircraft “call-in” on the frequency 134.475 MHz, before entering the territory of the Slovak Republic at the PODAN point, the correct indication of direction was found, provided that the receiver remained switched on FM signal reception even with this AM signal reception. This fact was verified by us several times by broadcasting from the VERTEX VXA-710 hand-held radio station on the frequency of 123.45 MHz. This testing was the same as testing with the YAESU VX-6R radio. This experiment proved that our targeting/searching device could also target amplitude-modulated signals that transmit with sufficient transmission power.

By performing experimental measurements using the IDVOR device, the possibility of using the Doppler effect by relative displacement of the sensor (monopole of the IDVOR antenna system) was tested in real conditions; this serves to detect the direction of the radio signal propagation source quickly. Therefore, it is possible to use an arbitrarily selected high-frequency frequency of the transmitter placed on the UAV, which is activated in an emergency or after a UAV failure/accident. By tuning the receiving targeting device IDVOR to this frequency, it is possible to navigate the search team in unknown terrain directly to the source of this high-frequency signal, i.e., to the searched UAV.

## 4. Conclusions

The reasoning that led the authors to create the IDVOR targeting device is based on the assumption of the use of UAVs as collection devices or devices intended for transport and carrying of payloads. In the current worldwide situation, it is unacceptable for equipment serving a dedicated group of people to be misused by a third party, not only from an economic but also a strategic point of view. For this reason, it is necessary to create a system that can detect transmissions on any selected frequency and at the same time reliably target the signal and bring a search team to its source.

The created IDVOR targeting system combines the advantages of the long-used DVOR aircraft device and the compass sight. This allows an accurate course guide (for the searching team) in unknown terrain, which is required by the tactical UAV manufacturer. Conventional tracking systems working with the signal from GNSS satellites, their evaluation and map guidance (in the case of a mapped area) are mainly unusable in the event of unexpected attacks, such as the possibility of intentional interference or degradation of the signal, intentional guidance to the wrong location, etc. The IDVOR device created by us provides a solution to this situation, the transmission frequency of the UAV device is arbitrarily chosen; at the same time, it is known only to a small group of people operating in the given area. In case of accident or failure UAV, the signal is captured only by the tuned receiving targeting device IDVOR, which the author’s team tested in real conditions; in a more mountainous area, but also on a flat meadow surrounded by trees.

The designed, constructed and successfully tested IDVOR device is thus able to provide an alternative way of solving a search in unknown terrain, or it could be used as a supplement method/device for other search and location means due to its ability to determine the direction of the transmitted signal. Its use is also possible in other applications, as desired by the end-user. For example, it may be the targeting and tracing of crashed UAVs serving as additional vehicles for HEMS, and we addressed the issue in recently published articles based on the societal demand of company ATE (Air-Transport Europe) [35]. 

From Table 3, it is clear that amateur constructions of Doppler direction finders cannot achieve the accuracy of the commercial devices. Nevertheless, these amateur constructions can reach the parameters of some commercial devices and thus fulfil simple tasks such as the proposed scenario in this article. Therefore, when comparing professional/commercial and amateur devices, the IDVOR can be found as a reasonable alternative for lost/crashed UAV targeting.

The presented experimental device and method have some advantages and disadvantages to be considered and discussed. It does not represent the final state or final product, as it is still under development. The next stage of development will be the testing of a higher number of antennas (eight, sixteen, etc.) in order to increase the accuracy of the IDVOR targeting system. We expect that the eight-antennas configuration can bring us an accuracy of 2.5°.

The companies that use high-value UAVs to intervene in unknown terrain create a societal requirement to address issues related to the localisation and targeting of UAVs that serve the company’s needs. Thus, they avoid financial losses, technical–structural losses and can primarily protect their property.

## Figures and Tables

**Table 1:** Counter drone detection methods.

Detection Method	Range	Advantages	Disadvantages
Acoustic sensors	Short distances (300 m–500 m)	Detection of even low-flying UAVs; entirely passive; fast sensor placement.	Excessive ambient noise reduces the likelihood of detection.
Optical sensors (IR, thermal imaging)	Usually, medium to short distances	Visual observation of UAV with the possibility of payload detection.	High detection error rate (e.g., birds). Low performance in poor lighting and weather.
Radars	Long distances (1 m–10 km)	High accuracy; the ability to detect multiple UAVs at once; independence of weather and time of day.	Active system; requires a license; cannot distinguish birds from UAVs.
RF Analysers	Medium to short distances	Low price; passive system; the possibility of triangulation of transmitter and UAV.	The need to use multiple receiving stations for triangulation; inability to detect passive UAVs.
Radio Direction Finders	Long to very long distances (1 m–50 km)	Low price; passive system; the possibility of triangulation of transmitter and UAV, accuracy from 2° to 5°.	The need to use multiple receiving stations for triangulation; inability to detect passive UAVs.

**Table 2:** Parameters for testing and evaluation of IDVOR.

RF Source for Targeting	GPS Coordinates	Real Bearing	Indicated Bearing	Error
OM0OUP	49.0640868° N 21.2560559° E	239°	236°	3°
OM0OVT	48.8512036° N 21.4929431° E	192°	191°	1°
PODAN	49.4129785° N 21.4392396° E	332°	331°	1°

**Table 3:** Comparison of parameters of commercial and amateur radio direction finders.

Radio Direction Finder	Accuracy	Number of Antennas	Frequency Range
WD-7200 HF/VHF/UHF Interferometer	2°	unknown	100 MHz–2 GHz
Mobile Radio Direction Finder (Doppler Systems)	2.5°	4	100 MHz–1 GHz
DF2020T RDF (GLOBAL TSCM GROUP)	5°	4	100 MHz–1 GHz
MFJ-5005 DDF W/GPS (MFJ Enterprises)	5°	4	100 MHz–1 GHz
RT-800 VTS RDF (RhoTheta)	2°	unknown	118 MHz–410 MHz
PA8W DDF (amateur)	5°	4	25 MHz–500 MHz
IDVOR (amateur)	5°	4	88 MHz–500 MHz

## Data Availability

Data sharing is not applicable.
